# Efficacy and safety of low- and high-intensity magnetic field therapies for orthopedic pain: a systematic review

**DOI:** 10.3389/fpain.2026.1760721

**Published:** 2026-04-30

**Authors:** Ludimila Dias Silva, Joelington Dias Batista, Jobson Dias Batista, Gabrielly Santos Pereira, Marcelo Lourenço da Silva

**Affiliations:** 1 Orthopain Pain Institute, Anápolis, GO, Brazil; 2 Neuropsychobiology and Motor Control Laboratory, Department of Health Sciences, Ribeirão Preto Medical School, University of São Paulo, Ribeirão Preto, São Paulo, Brazil; 3 Laboratory of Neuroscience, Neuromodulation and Study of Pain (LANNED), Federal University of Alfenas (UNIFAL-MG), Alfenas, MG, Brazil

**Keywords:** chronic low back pain, musculoskeletal pain, non-invasive neuromodulation, osteoarthritis, pulsed electromagnetic field therapy, repetitive peripheral magnetic stimulation

## Abstract

**Introduction:**

Orthopedic and musculoskeletal pain is a leading cause of disability worldwide, and many patients continue to experience suboptimal relief despite advances in pharmacological and physical therapies. Magnetic field–based interventions, including pulsed electromagnetic field therapy (PEMF) and repetitive peripheral magnetic stimulation (rPMS), have emerged as non-invasive approaches for pain modulation and functional improvement. This systematic review aimed to evaluate the efficacy and safety of low-intensity (PEMF) and high-intensity (rPMS) magnetic field therapies in adults with musculoskeletal conditions.

**Methods:**

Randomized controlled trials published between 2015 and 2025 were identified through searches in PubMed, Embase, Scopus, and Web of Science. Eligible studies compared PEMF or rPMS with sham, placebo, or standard care and reported outcomes related to pain intensity, functional performance, and safety.

**Results:**

Eight RCTs met the inclusion criteria. Most included studies reported reductions in pain and improvements in functional outcomes, including the Oswestry Disability Index and WOMAC. No serious adverse events were reported. PEMF was primarily linked to sustained analgesic and anti-inflammatory effects, whereas rPMS showed faster pain reduction, likely related to neuromuscular activation and modulation of descending inhibitory pathways. Some studies reported greater benefits using higher stimulation intensities or combining therapy with exercise.

**Discussion:**

Magnetic field therapies appear to be safe and well tolerated, with potential benefits for pain and function for the management of musculoskeletal pain. Their complementary mechanisms suggest potential clinical synergy; however, larger and more standardized trials are needed to establish optimal protocols and long-term effectiveness.

**Systematic Review Registration:**

https://www.crd.york.ac.uk/PROSPERO/view/CRD420251170876, PROSPERO CRD420251170876.

## 1 Introduction

Orthopedic and musculoskeletal pain represents one of the leading causes of physical disability in adults, exerting a substantial impact on quality of life and public health expenditures. Conditions such as osteoarthritis, low back pain, and postoperative pain rank among the most prevalent clinical diagnoses and often progress to chronic pain syndromes characterized by central sensitization, impaired functional performance, and psychosocial distress (1). Although conventional pharmacological and physiotherapeutic management provides partial relief, the search for safe and effective complementary therapies remains a clinical priority.

In recent years, magnetic field–based therapies, including pulsed electromagnetic field therapy (PEMF) and repetitive peripheral magnetic stimulation (rPMS), have emerged as promising non-invasive approaches for the modulation of pain and musculoskeletal function (2, 3). PEMF employs low-frequency (1–50 Hz), low-intensity magnetic fields that induce secondary electric currents within tissues, thereby influencing tissue regeneration, inflammatory processes, and neural conduction (4, 5). In contrast, rPMS deliver higher-intensity magnetic pulses capable of eliciting visible muscle contractions and inducing both peripheral and central neuromodulatory effects on nociceptive pathways (5, 6).

Both pulsed electromagnetic and repetitive magnetic stimulation share neurophysiological mechanisms based on the induction of electric fields in biological tissues, which can modulate neuronal excitability and promote synaptic plasticity (7). PEMF primarily acts on peripheral tissues, altering membrane potentials, ion fluxes (particularly calcium and sodium), and the expression of local inflammatory mediators such as prostaglandins and cytokines (8). These effects favor tissue repair, angiogenesis, and bone regeneration while attenuating peripheral sensitization.

Although repetitive transcranial magnetic stimulation (rTMS) has been shown to modulate corticospinal excitability and descending inhibitory pathways, these mechanisms are primarily derived from cortical stimulation paradigms and may not directly apply to peripheral magnetic stimulation modalities (7). Both modalities ultimately aim to restore the balance between neuronal excitation and inhibition, targeting central and peripheral mechanisms underlying pain chronification (9, 10).

Musculoskeletal pain of orthopedic origin—such as low back pain, discopathy, osteoarthritis, and postoperative pain syndromes—shares common pathophysiological mechanisms, including persistent local inflammation, peripheral and central hypersensitivity, and autonomic imbalance (8). PEMF exerts anti-inflammatory and analgesic actions by downregulating COX-2, IL-1β, and TNF-α expression, modulating voltage-gated ion channels, and enhancing microcirculation, thereby promoting tissue repair and pain relief in conditions such as osteoarthritis and delayed bone consolidation (6, 8).

Similarly, rPMS can attenuate cortical hyperexcitability and restore the function of disrupted corticospinal networks following musculoskeletal injury, surgery, or chronic pain. By activating serotonergic and noradrenergic descending pathways, rPMS contributes to sustained analgesia and improved motor recovery (8, 11).

Despite the increasing number of clinical trials investigating PEMF and rPMS in orthopedic contexts, results remain heterogeneous and fragmented. Variations in stimulation parameters, targeted conditions, and protocol duration hinder between-study comparison and limit the generalizability of findings. Moreover, prior reviews often focused on a single stimulation modality or included heterogeneous populations encompassing neuropathic or fibromyalgia-related pain rather than strictly musculoskeletal disorders.

In this context, a comprehensive synthesis of the most recent evidence is warranted. Therefore, the present systematic review aimed to critically evaluate the efficacy and safety of low-intensity (PEMF) and high-intensity (rPMS) magnetic field therapies for the treatment of orthopedic and musculoskeletal pain in adults, based on randomized controlled trials published over the past decade.

## 2 Materials and methods

### 2.1 Study design

This systematic review evaluates the efficacy and safety of low- and high-intensity magnetic field therapies for the management of orthopedic and musculoskeletal pain in adults.

The review follows the Preferred Reporting Items for Systematic Reviews and Meta-Analyses (PRISMA) 2020 guidelines and is prospectively registered in PROSPERO (CRD420251170876).

### 2.2 Eligibility criteria

Eligibility criteria are defined according to the PICO framework:

Population: Adults (≥18 years) with orthopedic or musculoskeletal pain conditions, including osteoarthritis, chronic low back or neck pain, tendinopathies, myofascial pain, and postoperative orthopedic pain.

Studies exclusively in pediatric or animal models are excluded.

Intervention: Clinical trials employing magnetic field therapies with clearly defined stimulation parameters (frequency, intensity, pulse shape, session duration, number of sessions, total treatment period).

Interventions are categorized as: Low-intensity PEMF (non-contractile stimulation) and higher-intensity rPMS (capable of inducing visible muscle contractions).

Comparison: Sham or placebo stimulation, usual care (e.g., pharmacological therapy, physiotherapy, or exercise), or other active interventions.

Primary outcomes: Pain intensity [Visual Analog Scale (VAS), Numeric Rating Scale (NRS), or comparable tools]; disability indices [Oswestry Disability Index (ODI), WOMAC, or Roland-Morris Disability Questionnaire (RMDQ)].

Secondary outcomes: Functional performance (Timed Up and Go, Lequesne Index), range of motion, quality of life (SF-36, EQ-5D), analgesic consumption, and adverse events or treatment tolerability.

Study design: Only randomized controlled trials (RCTs) or quasi-RCTs published in peer-reviewed journals are included.

Non-randomized trials, uncontrolled studies, reviews, or abstracts without full data are excluded.

### 2.3 Information sources

Searches were conducted in PubMed/MEDLINE, Embase, Scopus, Web of Science, Cochrane Central (CENTRAL), and ScienceDirect. The search covers publications from October 20, 2015 to October 20, 2025 and includes full-text articles in English. Additional records are identified by screening reference lists of included studies and relevant reviews and by consulting domain experts.

### 2.4 Search strategy

The electronic search strategy combined controlled vocabulary (e.g., MeSH/Emtree) and free-text keywords related to peripheral magnetic stimulation and muscle outcomes. Boolean operators AND/OR are applied to combine terms.

In PubMed, the strategy includes terms such as: (“pulsed electromagnetic field therapy” OR “PEMF” OR “electromagnetic field therapy” OR “repetitive peripheral magnetic stimulation” OR “rPMS” OR “peripheral magnetic stimulation”)AND (“musculoskeletal pain” OR “orthopedic pain” OR “low back pain” OR “osteoarthritis” OR “tendinopathy” OR “myofascial pain”) AND (“randomized controlled trial” OR randomized OR placebo OR sham). The strategy is adapted for other databases. Reference lists of all included studies and relevant reviews are screened to identify further eligible records.

### 2.5 Study selection

All identified records were imported into Rayyan for duplicate removal and screening. Two independent reviewers (GSP and MLS) screen titles and abstracts, followed by full-text assessment based on the eligibility criteria. Disagreements were resolved through discussion or by a third reviewer (LDS). The selection process is documented using a PRISMA 2020 flow diagram, with reasons for full-text exclusions recorded.

### 2.6 Risk of bias assessment

Risk of bias was assessed using the Cochrane RoB 2 tool for the primary pain outcome of each included study, considering the first post-intervention assessment time point for the main between-group comparison. When the primary pain outcome was not explicitly defined by the authors, the most clinically relevant pain-related outcome was selected. Domain-level judgments and supporting justifications for each study are provided in Supplementary Table S1. Each domain is classified as low risk, some concerns, or high risk. Visual summaries are created using the ROBVIS (Risk Of Bias Visualization) tool.

### 2.7 Data synthesis

A narrative synthesis was conducted to summarize all included studies according to pain condition, magnetic field intensity, and stimulation protocol. Although quantitative pooling using a random-effects model was initially planned, a meta-analysis was not performed due to substantial clinical and methodological heterogeneity across studies, including variability in PEMF parameters (such as frequency, intensity, and waveform), differences in clinical populations, and diversity in outcome measures.

Therefore, results were synthesized descriptively, with studies studies were grouped according to pain condition, type of magnetic stimulation (PEMF vs. rPMS), and intervention design (PEMF alone vs. PEMF combined with exercise or other therapies). This approach aimed to identify consistent patterns of effects, explore potential intensity–response relationships, and highlight key methodological limitations, including small sample sizes, heterogeneity of stimulation protocols, and the lack of long-term follow-up assessments.

## 3 Results

A systematic search conducted in PubMed, Embase, Scopus, Web of Science, Cochrane CENTRAL, PEDro, and ClinicalTrials.gov identified a total of 4,247 records. After the removal of 367 duplicate records, 3,880 studies remained for title and abstract screening.

During the screening phase, 3,775 records were excluded for not meeting the predefined inclusion criteria, primarily due to non-orthopedic populations, absence of magnetic field–based interventions, or non-randomized study designs.

A total of 105 full-text articles were then assessed for eligibility. Of these, 97 studies were excluded for reasons including non-randomized or uncontrolled designs, irrelevant interventions (e.g., static magnetic fields or electrical-only stimulation), and insufficient reporting of pain or functional outcomes.

Ultimately, eight randomized controlled trials (RCTs) met all inclusion criteria and were included in this systematic review.

**Figure 1:**
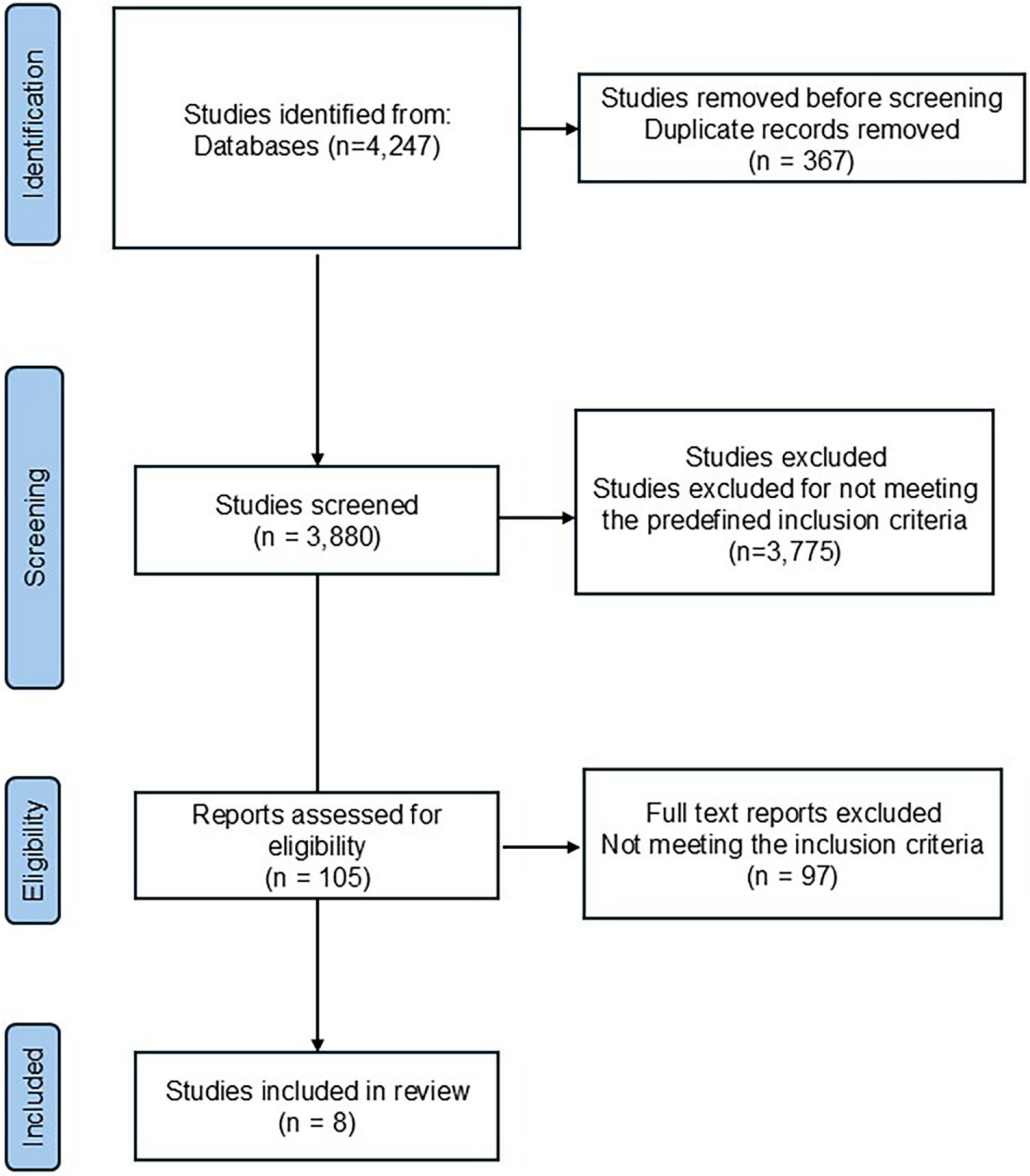
PRISMA flowchart.

These studies evaluated both low- and high-intensity magnetic field therapies—including pulsed electromagnetic field therapy (PEMF) and repetitive peripheral magnetic stimulation (rPMS)—for the treatment of conditions such as chronic low back pain, postoperative pain, and knee osteoarthritis. Across all included studies, interventions were generally well tolerated, with no reports of serious adverse events associated with magnetic field exposure. The PRISMA 2020 flow diagram summarizing the study selection process is presented in Figure 1.

### 3.1 Study selection and characteristics

From the 3,880 records screened, 105 full-text articles were assessed for eligibility after the exclusion of 3,775 records at the title and abstract stage. Among the full-text articles, 97 studies were excluded due to reasons such as non-randomized design, absence of appropriate control groups, inadequate reporting of stimulation parameters, or outcomes not related to pain or functional performance. Ultimately, eight RCTs met all inclusion criteria and were included in this systematic review (12–19). Collectively, these trials represent a decade of growing clinical interest in non-invasive magnetic field therapies—particularly pulsed electromagnetic field therapy (PEMF) and repetitive peripheral magnetic stimulation (rPMS)—as safe and effective adjuncts for managing orthopedic and musculoskeletal pain, including chronic low back pain, postoperative lumbar pain, and knee osteoarthritis.

Across the included trials, PEMF was the most frequently studied modality, typically applying low-frequency (1–200 Hz) and low-intensity (microtesla to millitesla range) fields via coil or pad applicators for 10–30 min per session over 2–4 weeks. In contrast, rPMS employed higher intensities (10–50 mT) with pulse trains up to 100 Hz, inducing visible muscle contractions and acute analgesia (17). It is important to note that reported intensity values varied substantially across studies and likely reflect device-specific output parameters rather than standardized measures of magnetic flux density, limiting direct comparison across trials.

While stimulation parameters varied, most trials implemented double- or single-blind sham-controlled designs, ensuring internal validity and minimizing bias.

Pain reduction was a consistent finding: most studies reported reductions in VAS pain scores by approximately 2–3 points compared with control or sham (13, 15, 16, 18, 19), with one high-intensity study reporting a large effect size (Cohen's d = 1.56) in acute low back pain (17). Functional improvements were reported in several studies, with significant gains in the Oswestry Disability Index (ODI) and WOMAC function scores in five trials (12, 15, 16, 18, 19).

A summary of study design, patient characteristics, sample size, interventions, outcome assessments and findings are listed in Table 1.

**Table 1:** Study characteristics of the included studies.

Study	Condition	*n*	Device type	Frequency	Intensity	Pulse width	Waveform	Applicator/coil	Target region	Session Duration	Sessions/Total Dose	Sham Description
Taradaj et al., (15)	Chronic LBP	106	PEMF	50 Hz/195 Hz	10 mT/5 mT/49.2 µT	NR	Pulsed	Coil applicator	Lumbar spine	20 min	15 sessions/3 weeks	Sham device without active field
Hartard et al., (17)	Acute LBP	61	rPMS-like induction device	NR (0.3–250 MHz carrier)	50 mT	NR	Pulsed EM induction	Applicator pad	Lumbar region	10 min	3 sessions/3 days	Sham device with no active emission
Elshiwi et al., (16)	Chronic LBP	50	PEMF	50 Hz	∼2 mT (20 gauss)	NR	Pulsed	Coil pads	Lumbar spine	20 min	12 sessions/4 weeks	Device inactive (placebo)
Sorrell et al., (14)	Postoperative LBP	36	PEMF	27.12 MHz	NR	38–42 µs	Pulsed RF	Wearable applicator	Lumbar region	30 min (2×/day)	60 days	Sham identical device
Arneja et al., (12)	Chronic LBP	21	PEMF	0.92–7.7 Hz	3.3 × 10−⁸–3.4 × 10−⁷ G	NR	Pulsed	Coil device	Lumbar spine	60 min	5 sessions/2 weeks	Placebo device
Comino-Suárez et al., (18)	Knee OA	60	PEMF	NR	NR	NR	NR	Local applicator	Knee joint	NR	NR	Sham comparator
Bagnato et al., (13)	Knee OA	60	PEMF	Low-frequency (NR)	NR	NR	Pulsed	Wearable device	Knee	Continuous (12 h/day)	1 month	Sham wearable
Ehsan Hashemi et al., (19)	Knee OA	70	PEMF	NR	NR	NR	NR	Local applicator	Knee	30 min	15 sessions/3 weeks	Placebo-controlled

VAS, visual analog scale; ODI, Oswestry disability index; RMDQ, Roland-Morris disability questionnaire; ROM, range of motion; NPRS, numeric pain rating scale; BDI-II, Beck depression inventory II; PGIC, patient global impression of change; WOMAC, Western Ontario and McMaster Universities Osteoarthritis Index; TUG, timed up and go; PGA, patient global assessment; AEs, adverse events; NR, not reported; LBP, low back pain; OA, osteoarthritis; µT, microtesla; mT, millitesla; G, gauss; µs, microseconds.

### 3.2 Interventions

Despite methodological heterogeneity, all included studies shared the defining characteristic of delivering non-invasive, low-risk magnetic stimulation aimed at modulating peripheral nociceptive activity and musculoskeletal recovery.

PEMF protocols typically applied low-intensity magnetic fields (microtesla to millitesla range) with frequencies between 1 and 200 Hz, delivered for 10–30 min per session, totaling 10 to 20 sessions across 2–4 weeks. Devices included coil or pillow applicators targeting the lumbar spine or knee.

High-intensity rPMS protocols induced visible muscle contractions using field strengths up to 50 mT, pulse trains at 5–100 Hz, and session durations between 10 and 30 min. Most studies were double-blind or single-blind and included sham-controlled or placebo-controlled groups to ensure internal validity and minimize expectancy bias.

### 3.3 Primary outcomes

#### 3.3.1 Pain intensity and symptom reduction

All eight RCTs assessed pain intensity as a primary outcome. Seven studies used the Visual Analog Scale (VAS), while one employed the Numeric Pain Rating Scale (NPRS).

Significant reductions in pain intensity were reported in seven trials compared with sham or control conditions. In chronic low back pain, PEMF reduced VAS scores by 2–3 points after 3–4 weeks of treatment (15, 16). In postoperative pain, rPMS decreased pain intensity by more than 40% and improved patient satisfaction (14). In knee osteoarthritis, PEMF produced significant reductions in both VAS and WOMAC pain subscales (18, 19). High-intensity stimulation (≥10 mT) suggested greater short-term effects than low-intensity or sham conditions (15, 17).

#### 3.3.2 Functional outcomes

Seven studies assessed functional performance or disability indices, most commonly using the Oswestry Disability Index (ODI), Western Ontario and McMaster Universities Osteoarthritis Index (WOMAC), or Roland-Morris Disability Questionnaire (RMDQ).

Five trials demonstrated statistically significant functional improvement after magnetic stimulation. Low back pain studies reported reductions of 8–10 points on the ODI (15, 16), while knee osteoarthritis trials found clinically meaningful improvements in WOMAC function, stiffness, and mobility tests (13, 18, 19).

Combined therapies (PEMF+exercise) yielded superior results compared to monotherapy, indicating potential synergistic effects between neuromodulation and active rehabilitation.

#### 3.3.3 Secondary outcomes and mechanistic findings

All included RCTs reported excellent tolerability and no serious adverse events. Minor, transient discomfort or muscle twitching occurred in isolated cases but resolved spontaneously. No participants withdrew due to adverse effects.

Three studies explicitly stated “no adverse events,” and the remainder reported general safety with ethics approval and informed consent documentation. Collectively, the findings confirm the favorable safety profile of both PEMF and rPMS in musculoskeletal rehabilitation.

#### 3.3.4 Safety and adverse events

All eight included RCTs reported safety or tolerability outcomes. Adverse events were generally assessed through patient self-report, clinical monitoring, or standardized follow-up evaluations, although definitions and reporting methods varied across studies.

No serious adverse events were reported in any of the included trials. Minor and transient effects—such as mild warmth, tingling, or muscle twitching—were reported in a small proportion of participants and resolved spontaneously without intervention.

Importantly, no study reported withdrawals due to adverse events, and overall adherence to the interventions was high. Three studies explicitly stated the absence of adverse events, while the remaining trials reported favorable safety profiles without clinically significant complications.

These findings suggest that both PEMF and rPMS are safe and well tolerated in adult populations with musculoskeletal conditions, although the heterogeneity in reporting methods highlights the need for standardized safety assessment in future trials.

#### 3.3.5 Limitations

Several methodological factors limit the generalizability of the findings:

1. Small sample sizes (*n* = 21–106) reduced statistical power;
2. Heterogeneity in intensity, frequency, and pulse characteristics precluded formal meta-analysis;
3. Short follow-up periods (≤3 months) limited conclusions on long-term efficacy;
4. Inconsistent sham design in some trials may have introduced bias; and
5. Incomplete reporting of coil geometry and magnetic flux density hindered reproducibility.

#### 3.3.6 Risk of bias

**Figure 2:**
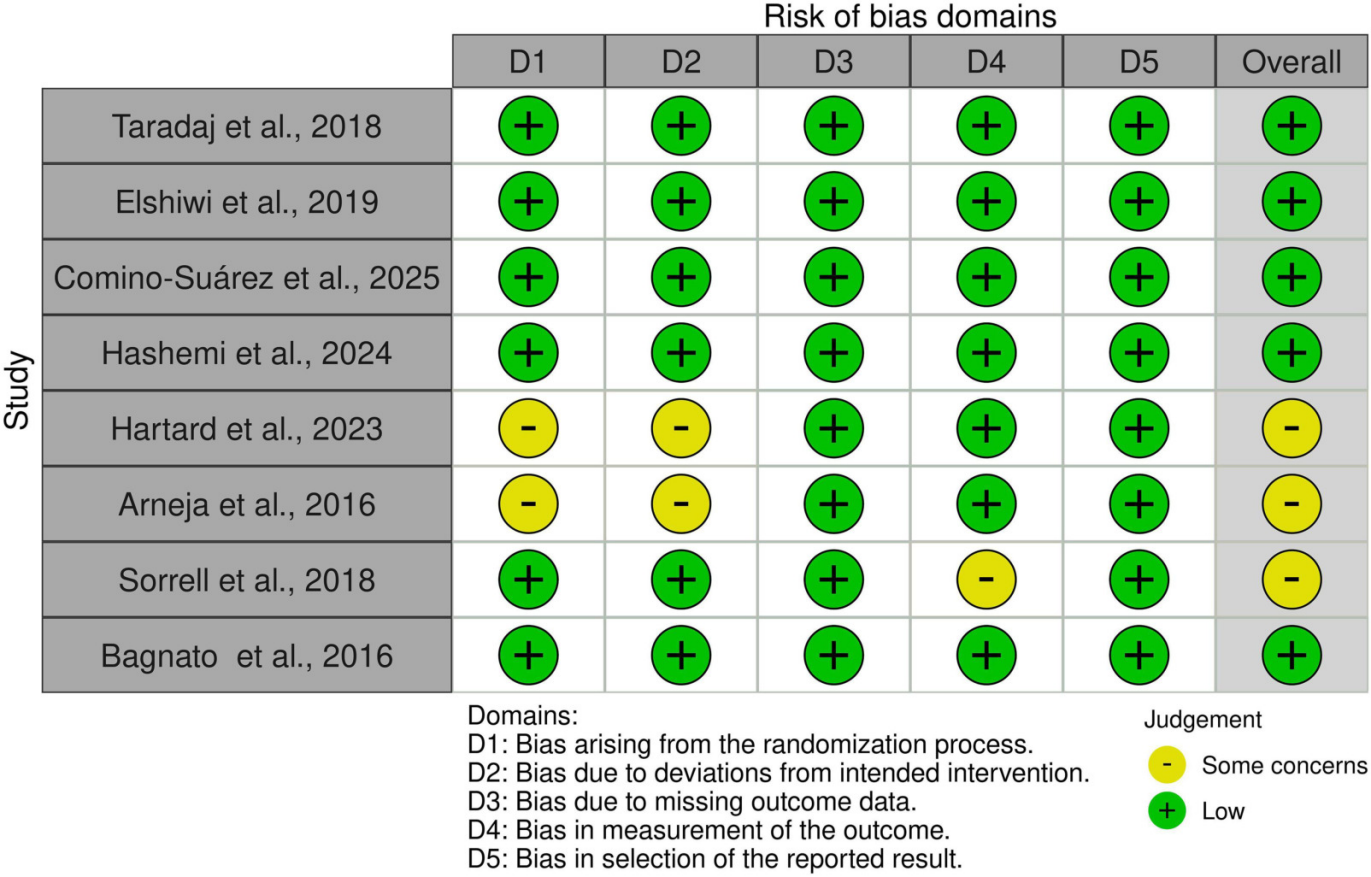
Evaluation of the different risk factors for bias from the studies included in this systematic review.

Risk of bias was assessed using the Cochrane RoB 2.0 tool. Most studies presented low or moderate risk across domains, demonstrating adequate randomization, allocation concealment, and blinded assessment. The Robvis figure (Figure 2) provides a visual summary of the judgments, while detailed domain-level assessments and study-specific justifications are presented in Supplementary Table S1.

Four RCTs (13, 15, 16, 18, 19) were judged low risk of bias with complete follow-up and appropriate sham controls. Two studies (12, 17) had some concerns due to limited reporting of allocation procedures, while one (14) exhibited some concerns related to selective reporting of functional outcomes.

Overall, no study presented high risk in outcome measurement or selective reporting, and the methodological quality of the evidence base was considered moderate to high, supporting the reliability of conclusions regarding the analgesic efficacy and safety of magnetic field therapies in orthopedic pain.

## 4 Discussion

The findings of this systematic review demonstrate that magnetic field therapies, both low-intensity pulsed electromagnetic field therapy (PEMF) and high-intensity repetitive peripheral magnetic stimulation (rPMS), appear to be effective and safe for the management of orthopedic and musculoskeletal pain in adults. Reductions in pain intensity were reported across studies such as chronic low back pain, postoperative pain, and knee osteoarthritis. Functional improvements were also reported in disability indices, including the Oswestry Disability Index (ODI) and the Western Ontario and McMaster Universities Osteoarthritis Index (WOMAC) (13, 15, 16, 18, 19). These findings support the role of magnetic stimulation as a non-invasive adjunct to conventional pain management (6, 8, 9).

Low-intensity PEMF, typically delivering micro- to millitesla fields at frequencies between 1 and 200 Hz, demonstrated efficacy primarily in medium-term pain relief and functional recovery. Its effects are mainly attributed to peripheral mechanisms, including modulation of inflammatory mediators, regulation of ion channels, and improvement of local microcirculation. These processes contribute to tissue repair, angiogenesis, and attenuation of peripheral sensitization, explaining the clinical benefits observed in osteoarthritis and degenerative lumbar conditions (3, 8). Studies combining PEMF with exercise or higher cumulative doses tended to show better outcomes, suggesting a possible dose–response relationship (15, 18).

High-intensity rPMS, may indirectly engage central mechanisms, including descending inhibitory pathways involving the periaqueductal gray and rostral ventromedial medulla, although such effects are inferred from broader neuromodulation literature and remain to be directly demonstrated in peripheral stimulation contexts (20). In the study by Hartard et al. (17), rPMS produced a large analgesic effect (Cohen's d = 1.56) in acute low back pain. This suggests that higher intensities may lead to faster and stronger short-term analgesia, although long-term effects remains to be established.

An additional source of heterogeneity relates to the definition and reporting of stimulation intensity. Across studies, magnetic field strength was inconsistently reported, with values expressed in different units (e.g., mT, T, or device-specific settings) and often without clarification regarding peak vs. average output or measurement location (e.g., coil surface vs. target tissue). As a result, direct comparison of intensity levels across studies is challenging. In this review, interventions were therefore classified based on functional characteristics—namely whether stimulation induced visible muscle contraction—rather than relying on fixed intensity thresholds. This approach is more consistent with current reporting practices and better reflects the physiological effects of PEMF and rPMS.

The observed analgesic efficacy of both modalities can be explained by their shared ability to modulate the excitation–inhibition balance in the nervous system. By reducing peripheral hyperexcitability and central sensitization, magnetic stimulation may help restore more stable neural activity (8, 11). In addition, rPMS appears to activate serotonergic and noradrenergic descending pathways, which may contribute to sustained analgesia, particularly in postoperative and spine-related pain (20).

The mechanisms underlying PEMF and rPMS are likely distinct and operate at different physiological levels. PEMF is primarily associated with peripheral effects, including modulation of inflammatory mediators, ion channel activity, and tissue repair processes. In contrast, rPMS induces neuromuscular activation through electromagnetic induction and may produce secondary central effects via afferent input and spinal–supraspinal interactions.

Substantial clinical heterogeneity was observed across the included studies, particularly regarding pain condition and chronicity. The review included chronic low back pain, knee osteoarthritis, and postoperative pain, which differ in underlying mechanisms and temporal profiles. Chronic conditions are more strongly associated with central sensitization and persistent inflammation, whereas acute pain is mainly driven by peripheral nociceptive input. These differences may influence treatment response. High-intensity rPMS appears to provide faster effects in acute pain, while PEMF may offer more sustained benefits in chronic conditions. Therefore, combining these conditions without stratification may limit interpretability and mask condition-specific effects (8, 11, 17).

Beyond symptom relief, magnetic stimulation may influence biomechanical and neuromuscular outcomes. rPMS has been associated with improved muscle activation and postural control, whereas PEMF appears to enhance microvascular perfusion and tissue oxygenation (17, 20). These complementary mechanisms suggest that magnetic therapies may support both pain reduction and functional recovery, which is particularly relevant in orthopedic rehabilitation.

Regarding tolerability and safety, all included RCTs reported excellent profiles with no serious adverse events. Reported side effects were mild and transient, such as warmth or muscle twitching, and did not lead to treatment discontinuation (12, 14). These findings support the feasibility of repeated sessions in clinical settings.

When comparing modalities, PMS appears to produce faster short-term analgesia, whereas PEMF may promote more sustained functional and tissue-level improvements. This difference likely reflects distinct mechanisms, with rPMS acting on neuromuscular excitability and central pathways, and PEMF targeting peripheral inflammation and regeneration. These approaches may be complementary and could be combined with exercise to optimize rehabilitation outcomes.

Despite encouraging results, several limitations must be acknowledged. Most trials included small samples (*n* = 21–106), short intervention periods (2–4 weeks), and limited follow-up, restricting conclusions about long-term efficacy. The heterogeneity in protocols also prevented meta-analysis, reinforcing the need for more standardized designs.

Future research should focus on dose optimization, identifying the most effective intensity, frequency, and treatment duration for specific conditions. The integration of neurophysiological biomarkers, such as EMG, fMRI, or MEPs, may help clarify mechanisms and personalize treatment approaches. Long-term studies assessing durability of effects and quality of life are also needed. It should be noted that mechanistic interpretations are partly based on indirect evidence from related neuromodulation techniques, and direct mechanistic studies in orthopedic populations remain limited.

In summary, this systematic review provides convergent evidence that low- and high-intensity magnetic field therapies are effective, safe, and well tolerated for orthopedic pain. rPMS offers rapid analgesia, whereas PEMF supports sustained recovery and tissue modulation. Together, these modalities represent promising tools for integration into evidence-based rehabilitation protocols.

## Data Availability

The original contributions presented in the study are included in the article/Supplementary Material, further inquiries can be directed to the corresponding author.
