# Supplementary material for: Efficacy and safety of low- and high-intensity magnetic field therapies for orthopedic pain: a systematic review
Source: Front Pain Res (Lausanne). 2026 Apr 30;7:1760721. doi: 10.3389/fpain.2026.1760721 (PMC13171806; doi:10.3389/fpain.2026.1760721)
Supplement: Supplementary file 1 [file Table1.docx]

**Supplementary Table S1.** Domain-level RoB 2 judgments and justifications

| **Study** | **Outcome assessed** | **Time point** | **Randomization process** | **Deviations from intended interventions** | **Missing outcome data** | **Measurement of outcome** | **Selection of reported result** | **Overall risk** | **Justification** |
| --- | --- | --- | --- | --- | --- | --- | --- | --- | --- |
| Taradaj et al., 2018 | VAS pain | Post-treatment (3 weeks) | Low risk | Some concerns | Low risk | Low risk | Some concerns | Some concerns | Allocation concealment unclear; otherwise well conducted |
| Hartard et al., 2023 | VAS pain | Post-treatment (3 days) | Some concerns | Low risk | Low risk | Low risk | Low risk | Some concerns | Limited reporting of randomization procedures |
| Elshiwi et al., 2019 | VAS pain | Post-treatment (4 weeks) | Low risk | Low risk | Low risk | Low risk | Low risk | Low risk | Well-described randomization and blinding |
| Sorrell et al., 2018 | NPRS pain | Post-treatment (60 days) | Some concerns | Some concerns | Low risk | Low risk | Some concerns | Some concerns | Selective reporting of outcomes and unclear allocation |
| Arneja et al., 2016 | VAS pain | Post-treatment (2 weeks) | Some concerns | Low risk | Low risk | Low risk | Some concerns | Some concerns | Small sample and limited methodological detail |
| Comino-Suárez et al., 2025 | VAS pain | Post-treatment (4 months) | Low risk | Low risk | Low risk | Low risk | Low risk | Low risk | Robust sham-controlled design |
| Bagnato et al., 2016 | VAS pain | Post-treatment (1 month) | Low risk | Low risk | Low risk | Low risk | Low risk | Low risk | Double-blind design with adequate reporting |
| Hashemi et al., 2025 | VAS pain | Post-treatment (7 weeks) | Low risk | Low risk | Low risk | Low risk | Low risk | Low risk | Adequate methodology and reporting |
